# Toxicology of Metals and Metalloids

**DOI:** 10.1155/2014/253738

**Published:** 2014-05-22

**Authors:** Fernando Barbosa Júnior, Marcelo Farina, Susana Viegas, Wilma De Grava Kempinas

**Affiliations:** ^1^Departamento de Análises Clínicas, Toxicológicas e Bromatológicas, Faculdade de Ciências Farmacêuticas de Ribeirão Preto, Universidade de São Paulo, 14040903 Ribeirão Preto, SP, Brazil; ^2^Departamento de Bioquímica, Centro de Ciências Biológicas, Universidade Federal de Santa Catarina, 88040900 Florianópolis, SC, Brazil; ^3^Grupo de Investigação Ambiente e Saúde, Escola Superior de Tecnologia da Saúde de Lisboa and Instituto Politécnico de Lisboa, 1990-096 Lisboa, Portugal; ^4^Departamento de Morfologia, Instituto de Biociências de Botucatu, Universidade Estadual Paulista, 18618970 Botucatu, SP, Brazil

Metal toxicology is one of the oldest areas of study of toxicology and one of the oldest environmental problems. Metals and metalloids are toxic elements at the top of the priority list of hazardous substances of the Agency for Toxic Substances and Disease Registry (ATSDR). However, several gaps of knowledge still exist that are related to their toxicity, mainly concerning the mechanisms of action. This special issue affords the opportunity to bring together the results of nine papers covering several aspects of the toxicology of metals and metalloids in* in vitro* and* in vivo* experimental models, as well as in exposed populations.

The molecular mechanisms mediating manganese- (Mn-) induced neurotoxicity, particularly in the immature central nervous system, are not completely understood. Based on that, T. V. Peres et al. suggested that altered intracellular MAPKs signaling pathways may represent an early event concerning the effects of Mn exposure in the immature brain.

*In vitro* cytotoxicity of *β*-SiC nanowires (ceramic material with a potential use as hard tissue replacement) was investigated by W. Xie et al. The authors have found that 100 nm long SiC nanowires increased oxidative stress in MC3T3-E1 cells, as determined by the concentrations of MDA (as a marker of lipid peroxidation) and 8-OHdG (indicator of oxidative DNA damage). Moreover, after treatment with 100 nm long SiC nanowires, the mitochondria were swelled and disintegrated, and the production of ATP and the total oxygen uptake were also decreased significantly.

G. Espinosa-Reyes et al. assessed the impact of mining activities on biotic communities within the District of Villa de la Paz in Mexico. Authors have observed that the concentrations of As and Pb in soil were higher than the Mexican's regulations for urban or agricultural areas.

Many phytoremediation technologies have been used for the remediation of metal polluted areas. This is a low cost process with several distinct advantages including improvement of the soil quality, cost-effective and technically feasible process, plants which serve as sufficient biomass for rapid remediation, promoting high rhizosphere activity, and finally restoration in a reasonable time frame. Based on that, M. Sabeen et al. showed the potential of* Arundo donax* L. for phytoextraction of cadmium (Cd) from contaminated soil and water.

T. S. Gonçalves et al. evaluated the cytotoxicity induced by orthodontic bands through survival tests on* Saccharomyces cerevisiae*, a microorganism that presents several genetic and biochemical characteristics similar to human cells. Three groups of bands were evaluated: silver soldered (SSB), laser soldered (LSB), and bands without any solder (WSB). Authors found SSBs to be cytotoxic, whilst LSBs were not, confirming that laser soldering may be a more biocompatible alternative for use in connecting wires to orthodontic appliances.

The chemical effects of uranium in rats following a chronic ingestion were investigated by I. Dublineau and coworkers. Biochemical and hematological indicators were measured and several different types of investigations (molecular, functional, and structural) were conducted in different organs. The specific sensitivity of the organs to uranium was deduced from nondeleterious biological effects, with the following thresholds (in mg/L): 0.2 for brain, >2 for liver, >10 for kidneys, and >20 for intestine, indicating a no-observed-adverse-effect level (NOAEL) threshold for uranium superior to 120 mg/L. Based on the chemical uranium toxicity, the tolerable daily intake calculation yields a guideline value for humans of 1350 g/L. This value was higher than the WHO value of 30 g/L, indicating that this WHO guideline for uranium content in drinking water is very protective and might be reconsidered.

Methylmercury (MeHg) is one of the most poisonous environmental contaminants, causing toxic effects in humans and experimental animals. Then, potential protective effects of several nutrients against MeHg-induced toxicity have been evaluated. C. L. Dalla Corte et al. investigate the efficacy of diphenyl diselenide [(PhSe)2] in attenuating methylmercury- (MeHg-) induced toxicity in rats. Cotreatment with (PhSe)2 protected hepatic and cerebral mitochondrial thiols from depletion by MeHg but failed to completely reverse MeHg's effect on hepatic and cerebral mitochondrial dysfunction or hepatic, cerebral, and renal inhibition of TrxR activity. Additionally, the cotreatment with (PhSe)2 increased Hg accumulation in the liver (50.5%) and brain (49.4%) and increased the MeHg-induced motor deficits and body-weight loss. Their results indicate that (PhSe)2 can increase Hg body burden as well as the neurotoxic effects induced by MeHg exposure in rats. On the other hand, R. Frenedoso da Silva et al. observed that Maná-cubiu (*Solanum sessiliflorum* Dunal), a native fruit from the Amazon rich in iron, zinc, niacin, pectin, and citric acid, when coadministered with MeHg minimizes the damages caused by the exposure to MeHg on sperm quantity and quality and the histological aspect of the testis and epididymis of rats.

A. Á. Soares de Oliveira et al. evaluated the effects of polymorphisms in glutathione- (GSH-) related genes (GSTM1, GSTT1, GSTP1, GCLM, and GCLC) in the distribution of Hg in the blood compartments in humans exposed to methylmercury (MeHg). They observed that GSH-related polymorphisms might change the metabolism of MeHg by modifying the distribution of mercury species (inorganic mercury and methylmercury) in the plasma compartment and the partitioning between Hg in plasma and in blood.

We hope that the new findings on the toxic effects of metals and metalloids presented here can contribute to environmental and health policies, leading to a safer planet.

